# Is orthodontic treatment associated with changes in self-esteem during adolescence? A longitudinal study

**DOI:** 10.1177/14653125211006113

**Published:** 2021-04-16

**Authors:** Srichitra Vulugundam, Lucas Guimarães Abreu, Eduardo Bernabé

**Affiliations:** 1 Faculty of Dentistry, Oral & Craniofacial Sciences, King’s College London, London, UK; 2 Department of Child’s and Adolescent’s Oral Health, Universidade Federal de Minas Gerais, Belo Horizonte, Brazil

**Keywords:** psychological aspects of orthodontics, health services and quality of life aspects, epidemiology in orthodontics (including occlusal indices), quality of life and orthodontics

## Abstract

**Objective:**

This study explored the association between history of orthodontic treatment and changes in self-esteem among British adolescents.

**Design:**

Birth cohort study.

**Setting:**

United Kingdom.

**Participants:**

Data from 2600 participants of the British Cohort Study were analysed.

**Methods:**

Participants completed the Lawrence Self-Esteem Questionnaire (LAWSEQ) at the ages of 10 and 16 years. The change in LAWSEQ score over six years was the outcome. Participants were divided into two groups according to parental reports of orthodontic treatment at the same ages. The association between history of orthodontic treatment and six-year changes in LAWSEQ score was tested in linear regression models adjusting for demographic factors (adolescents’ sex and ethnicity), family socioeconomic status (parental social class and mothers’ education), perceived orthodontic treatment need and baseline LAWSEQ score.

**Results:**

According to parental reports, 8% of participants had a history of orthodontic treatment. The mean LAWSEQ score at baseline was 13.7 (95% confidence interval [CI] 13.6–13.9) and increased significantly over time by 1.7 units (95% CI 1.5–1.9). Orthodontic treatment history was positively, albeit not significantly, associated with change in LAWSEQ score (coefficient: 0.08, 95% CI –0.56 to 0.72). The direction of the association reversed but remained non-significant after adjustment for all confounders (coefficient: –0.19; 95% CI –0.68 to 0.30). Improvements in self-esteem were positively associated with mothers’ education (coefficient: 0.85; 95% CI 0.09–1.61) and negatively associated with self-esteem at baseline (coefficient –0.79; 95% CI –0.82 to –0.75).

**Conclusion:**

This six-year longitudinal study provided no evidence for an association between history of orthodontic treatment and changes in self-esteem during adolescence.

## Introduction

Orthodontic treatment aims to correct malocclusion, which can lead to improvements in masticatory function and dental aesthetics (Benson et al., 2015; Kang and Kang, 2014). Orthodontic treatment can also provide more global benefits in terms of improved psychological and social wellbeing (Ferrando-Magraner et al., 2019; Javidi et al., 2017; Kiyak, 2008; Zhou et al., 2014). A key aspect of psychological wellbeing is self-esteem, a multidimensional concept defined as one’s awareness of the self (Lawrence, 1981; Rosenberg et al., 1995). There is evidence that average self-esteem increases during late childhood and adolescence (Erol and Orth, 2011; Orth et al., 2018), which is usually the period when orthodontic treatment is sought. In the 2013 Children’s Dental Health Survey, 3%, 21% and 16% of 8-, 12- and 15-year-old British children were under orthodontic treatment (Tsakos et al., 2015).

An early review suggested that psychological wellbeing as represented by self-esteem does not seem to be affected by orthodontic treatment (Kiyak, 2008). Longitudinal studies in this area are scarce and their results are highly controversial. In two studies, patients were followed over the course of orthodontic treatment. In the first study, 61 adult patients from four private orthodontic clinics in England were recruited and a significant improvement in the Rosenberg Self-Esteem Scale (RSES) score from baseline to the end of treatment was found (Johal et al., 2015). In the second study, 118 adolescents aged 11–16 years from a university hospital in Belgium were recruited and changes in self-esteem over time, measured with the Harter’s Self Perceptions Profile for Adolescents, were not observed (Avontroodt et al., 2020). Both studies have limitations, including the small sample size, the lack of external control group and no adjustment for potential confounders, such as prior orthodontic treatment need and socioeconomic position. In two population-based studies, larger samples and longer follow-up periods have been used. The first was a 20-year longitudinal study of 254 Welsh children aged 11–12 years showing an association between orthodontic treatment and the RSES score at follow-up, which was fully accounted for by the RSES score at baseline (Kenealy et al., 2007; Shaw et al., 2007). The second was an 18-year longitudinal study with 427 Australian children aged 13 years showing that the receipt of orthodontic treatment with fixed appliances was associated with lower RSES scores at follow-up, after adjustment for orthodontic treatment need and income (Arrow et al., 2011). However, no adjustment for baseline RSES score was carried out.

Given the scarcity of studies in this important research area, a study addressing some of the limitations of previous studies was set up to fill this gap in knowledge. Therefore, the aim of the present study was to evaluate the association between history of orthodontic treatment and changes in self-esteem among British adolescents. It was hypothesised that orthodontic treatment is positively associated with changes in self-esteem during adolescence.

## Participants and methods

### Study population

The present study used historical data from the British Cohort Study 1970 (BCS70), an ongoing population-based study collecting data on various social and health aspects as cohort members move from childhood to adolescence to adulthood. The BCS70 recruited 17,196 children born within a week of April 1970 in England, Wales and Scotland (Elliott and Shepherd, 2006). Cohort members have been followed until they die or permanently emigrate from Great Britain. To date, 10 sweeps have been conducted, and the most recent sweep was carried out in 2016, when cohort members were 46 years old.

For inclusion in the study sample for the present research, cohort members should have participated in sweeps 3 (aged 10 years in 1980) and 4 (aged 16 years in 1986), and have complete information on orthodontic treatment, self-esteem and confounders. A total of 14,875 (90.1%) and 11,622 (70.4%) cohort members participated in sweeps 3 and 4, respectively (Mostafa and Wiggins, 2015).

### Measures

The outcome measure was adolescents’ self-esteem, which was measured with the Lawrence’s Self-Esteem Questionnaire (LAWSEQ) (Lawrence, 1981, 2006), at the ages of 10 and 16 years. The LAWSEQ was chosen because it had been recently devised by a former Chief Educational Psychologist of Somerset London Education Authority for use with British adolescents. All the 16 LAWSEQ items (of which four are distractors) were answered at age 10 years, with three response options (yes, no and don’t know). At age 16 years, only 10 of the 16 items with similar response options were answered. Excluded were the four distractor items (which are not used during scoring) as well as the items ‘Do you often feel sad because you have nobody to play with at school?’ and ‘When you have to say things in front of other children do you usually feel foolish?’ For the 10 common items, the answer for the positive ones were coded as yes (2), don’t know (1) and no (0), and the answers for the negative ones were reverse coded. The change in adolescents’ LAWSEQ score from age 10 to age 16 was used as the outcome measure, which was estimated by subtracting the score at age 10 years from that at age 16 years. Missing responses were imputed with the participants’ mean across all available LAWSEQ items. However, individuals with five or more missing responses were excluded. The LAWSEQ is a valid and reliable measure for use with children and adolescents (Hart, 1985; Lawrence, 2006). The Cronbach alpha in the study sample was 0.631 at age 10 years and 0.673 at age 16 years.

History of orthodontic treatment was the exposure of interest. Data on orthodontic treatment history were collected at ages 10 and 16 years. At age 10 years, parents answered the question ‘Does your child wear braces?’ and at age 16 years, parents answered the question ‘Does your teenager wear dental braces?’, with three answer options (yes, no and don’t know). Participants with a positive response to any of the two questions were classified as having a history of orthodontic treatment and those with negative responses to both questions were considered as having no history of orthodontic treatment.

Demographic factors (adolescents’ sex and ethnicity), family socioeconomic position (parental social class and mothers’ education) and perceived need for orthodontic treatment were the confounders considered for this study. Parental social class was determined based on parents’ occupations following the Registrar General’s Social Class (RGSC) classification system. The six RGSC groups were combined into three groups as follows: professional (I) and intermediate occupations (II) were the top class (I/II); skilled non-manual (III-NM) and skilled manual occupations (III-M) were the medium class (IIINM-M); and partly skilled (IV) and unskilled occupations (V) were the bottom class (IV/V). The highest social class of any parent was chosen to represent parental social class. Mothers’ education was recoded as no qualification, below degree level, and at degree level or above. Perceived need for orthodontic treatment was indicated by whether the child had had a consultation with an orthodontist (either in public or private dental services) in the past year, which was reported by parents in sweep 3 (age 10 years).

### Data analysis

All analyses were conducted in Stata version 16 (StataCorp, College Station, TX, USA). First, participants with complete data were compared with individuals excluded because of missing values, using the chi-square test for categorical variables (adolescents’ sex, adolescents’ ethnicity, mother’s education, parental social class, perceived orthodontic need and adolescents’ history of orthodontic treatment) and the t-test for numerical variables (self-esteem scores at ages 10 and 16 years, and change in LAWSEQ score). LAWSEQ scores were normally distributed (Shapiro–Wilk test, all *P* > 0.05). Then, participants with and without orthodontic treatment history were compared in terms of LAWSEQ scores (at age 10 years, age 16 years and change in LAWSEQ score) and confounders (adolescents’ sex, adolescents’ ethnicity, mother’s education, parental social class and perceived orthodontic treatment need). As before, the chi-square test and the t-test were used for comparing categorical and numerical variables, respectively.

The crude and adjusted associations between history of orthodontic treatment and changes in the LAWSEQ score were tested using linear regression as the outcome measure was a numerical variable with normal distribution. Therefore, regression coefficients with 95% confidence intervals (CI) were reported as the measure of association. The adjusted regression model included controls for demographic factors (adolescents’ sex and adolescents’ ethnicity), family socioeconomic position (parental social class and mothers’ education) and perceived need for orthodontic treatment. A possible interaction between orthodontic treatment history and perceived orthodontic treatment was tested by adding their multiplication as an additional predictor to the regression model.

## Results

A total of 4060 participants completed the LAWSEQ at ages 10 and 16 years. Of them, 2815 had information on history of orthodontic treatment. A total of 215 were excluded because of missing values on confounders (adolescents’ ethnicity = 2, mothers’ education = 129, parental social class = 71, and perceived orthodontic need = 23). There were significant differences between participants in the study sample and individuals excluded due to missing values in terms of adolescents’ ethnicity (*P* = 0.040) and mothers’ education (*P* < 0.001). There was a significantly higher proportion of white participants (97.0% vs. 94.4%) and of adolescents whose mothers had some qualification (54.7% vs. 29.1%) in the study sample than in the group of excluded individuals. No differences between groups in terms of orthodontic treatment history and changes in LAWSEQ score were observed. In the study sample, the mean change in LAWSEQ score was 1.7 ± 4.5 points (range = −16 to 15.7) and 8% of adolescents had history of orthodontic treatment (Table 1).

**Table 1.** Comparison of adolescents in the study sample with those excluded because of missing values.

Covariates	Excluded (n = 215)	Study sample (n = 2600)	*P* value*
*Adolescent’s sex*			0.752
Male	88 (40.9)	1093 (42.0)	
Female	127 (59.1)	1507 (58.0)	
*Adolescent’s ethnicity*			0.040
White	201 (94.4)	2521 (97.0)	
Non-white	12 (5.6)	79 (3.0)	
*Mother’s education*			<0.001
No qualification	61 (70.9)	1178 (45.3)	
Below degree	20 (23.3)	1332 (51.2)	
Degree	5 (5.8)	90 (3.5)	
*Parental social class*			0.075
IV/V (lowest)	21 (14.6)	275 (10.6)	
III-N/III-M	77 (53.5)	1269 (48.8)	
I/II (highest)	46 (31.9)	1056 (40.6)	
*Perceived orthodontic need*			0.334
No need	191 (99.5)	2566 (98.7)	
In need	1 (0.5)	34 (1.3)	
*Adolescent’s orthodontic treatment history*			0.080
No treatment history	205 (95.3)	2393 (92.0)	
With treatment history	10 (4.7)	207 (8.0)	
Outcome measures			*P* value*
LAWSEQ score at age 10 years	13.4 ± 4.0	13.7 ± 3.8	0.255
LAWSEQ score at age 16 years	15.5 ± 3.5	15.4 ± 3.5	0.729
Change in self-esteem score	2.1 ± 4.9	1.7 ± 4.5	0.220

Values are given as n (%) or mean ± SD.

* Chi-square test was used to compare categorical variables and independent t-test to compare numerical variables.

LAWSEQ, Lawrence Self-Esteem Questionnaire; SD, standard deviation.

Significant differences between participants with and without orthodontic treatment history were found in relation to adolescents’ sex, mothers’ education and perceived orthodontic treatment need. There were significantly higher proportions of female participants, those with more educated mothers and those with perceived need for orthodontic treatment in the group with history than in the group without history of orthodontic treatment. However, there were no differences in the change in LAWSEQ score between groups (Table 2).

**Table 2.** Comparison of covariates and LAWSEQ scores between adolescents with and without history of orthodontic treatment.

Covariates	No treatment history (n = 2393)	With treatment history (n = 207)	*P* value*
*Adolescent’s sex*			0.001
Male	1028 (43.0)	65 (31.4)	
Female	1365 (57.0)	142 (68.6)	
*Adolescent’s ethnicity*			0.253
White	2323 (97.1)	198 (95.7)	
Non-white	70 (2.9)	9 (4.3)	
*Mother’s education*			0.013
No qualification	1104 (46.1)	74 (35.7)	
Below degree	1209 (50.5)	123 (59.4)	
Degree	80 (3.3)	10 (4.8)	
*Parental social class*			0.621
IV/V (lowest)	257 (10.7)	18 (8.7)	
III-N/III-M	1168 (48.8)	101 (48.8)	
I/II (highest)	968 (40.5)	88 (42.5)	
*Perceived orthodontic need*			<0.001
No need	2374 (99.2)	192 (92.8)	
In need	19 (0.8)	15 (7.2)	
Outcome measures			*P* value*
LAWSEQ score at age 10 years	13.7 ± 3.8	13.5 ± 3.7	0.403
LAWSEQ score at age 16 years	15.4 ± 3.5	15.3 ± 3.6	0.544
Change in self-esteem score	1.7 ± 4.5	1.8 ± 4.3	0.816

Values are given as n (%) or mean ± SD.

* Chi-square test was used to compare categorical variables and independent t-test to compare numerical variables.

LAWSEQ, Lawrence Self-Esteem Questionnaire; SD, standard deviation.

Table 3 shows the associations between orthodontic treatment history and changes in LAWSEQ score. The interaction between orthodontic treatment history and perceived orthodontic treatment need was not significant (*P* = 0.843) and was therefore not included in the final regression model. In the adjusted model, participants with orthodontic treatment history had a smaller increase in LAWSEQ score over time (coefficient = −0.19; 95% CI = −0.68 to 0.30) than those without orthodontic treatment history. However, this difference was not significant. Only mothers’ education and baseline self-esteem score were significantly associated with changes in adolescents’ LAWSEQ score in the adjusted model. Participants whose mothers had higher education had greater increases in LAWSEQ score (coefficient = 0.85; 95% CI = 0.09–1.61) than those whose mothers had no qualification. In addition, higher LAWSEQ scores at age 10 years were associated with reductions in LAWSEQ score over time (coefficient = −0.79; 95% CI = −0.82 to –0.75).

**Table 3.** Crude and adjusted associations between history of orthodontic treatment and changes in LAWSEQ score among adolescents (n = 2600).

Explanatory variables	Change in LAWSEQ score	Crude associations		Adjusted associations^*^	
		Coef.	[95% CI]	Coef.	[95% CI]
*Adolescents’ sex*					
Male	1.2 ± 4.4	0.00	[Reference]	0.00	[Reference]
Female	2.0 ± 4.6	0.85	[0.50–1.20]^†^	0.08	[–0.18 to 0.34]
*Adolescents’ ethnicity*					
White	1.7 ± 4.5	0.00	[Reference]	0.00	[Reference]
Non-white	1.5 ± 5.1	–0.22	[–1.23 to 0.79]	–0.39	[–1.16 to 0.38]
*Mothers’ education*					
No qualification	1.9 ± 4.6	0.00	[Reference]	0.00	[Reference]
Below degree	1.6 ± 4.5	–0.28	[–0.63 to 0.75]	0.30	[0.01–0.59]
Degree	1.3 ± 3.5	–0.59	[–1.55 to 0.38]	0.85	[0.09–1.61]^‡^
*Parental social class*					
IV/V (lowest)	1.8 ± 4.7	0.00	[Reference]	0.00	[Reference]
III-N/III-M	1.8 ± 4.6	0.00	[–0.59 to 0.58]	0.34	[–0.10 to 0.80]
I/II (highest)	1.5 ± 4.4	–0.35	[–0.94 to 0.25]	0.56	[0.08–1.04]
*Perceived orthodontic need*					
No need	1.7 ± 4.5	0.00	[Reference]	0.00	[Reference]
In need	2.1 ± 3.9	0.40	[–1.12 to 1.93]	0.46	[–0.71 to 1.63]
*Adolescents’ orthodontic treatment history*					
No treatment history	1.7 ± 4.5	0.00	[Reference]	0.00	[Reference]
With treatment history	1.8 ± 4.3	0.08	[–0.56 to 0.72]	–0.19	[–0.68 to 0.30]
*LAWSEQ score at age 10 years*	1.7 ± 4.5	–0.78	[–0.80 to –0.74]^†^	–0.79	[–0.82 to –0.75]^†^

Values are given as mean ± SD unless otherwise specified.

* Linear regression model was fitted and unstandardised regression coefficients were reported. The adjusted model included as predictors all variables shown in the table.

† *P* < 0.001.

‡ *P* < 0.05.

CI, confidence interval; Coef., coefficient; SD, standard deviation.

## Discussion

This retrospective study found no association between history of orthodontic treatment and change in self-esteem from the ages of 10 to 16 years. This finding was robust to adjustments for sociodemographic factors, perceived orthodontic treatment need and self-esteem at age 10. Against an increase in self-esteem scores over time, participants with a history of orthodontic treatment reported smaller (albeit non-significant) increases in self-esteem than those without history.

We can speculate on possible explanations for the non-significant findings. One could be related to the type of self-esteem being measured. Self-esteem measures, such as the Rosenberg scale and LAWSEQ are based on explicit (direct) self-evaluations (Pietschnig et al., 2018; Stieger et al., 2017). One criticism of explicit measures of self-esteem is that people are unable to evaluate themselves objectively (Pietschnig et al., 2018). In contrast, implicit (indirect) measures of self-esteem are based on automatic evaluations, of which individuals are unaware (De Houwer et al., 2009). Some argue that implicit and explicit self-esteem are completely independent of each other, implying that both must be assessed to obtain an overall representation of people’s self-esteem (Pietschnig et al., 2018; Stieger et al., 2017). Common implicit measures of self-esteem are the Initial Preference Task and the Implicit Association Test. Whether orthodontic treatment can impact implicit self-esteem is currently unknown.

An alternative explanation relates to the use of historical data and its relevance today when both orthodontic treatment and adolescents’ worlds, in terms of values and social expectations, have changed markedly. The BCS70 data used in this study correspond to the 1980s (i.e. participants were 10 years old in 1980). It is possible that people now seek orthodontic care for different reasons than in the past, particularly given the greater emphasis on body image and marketing strategies for cosmetic care as well as the impact of peers and social media on adolescents’ preferences. There is also some evidence that adolescent self-esteem has improved over time (Birndorf et al., 2005; Raustorp and Fröberg, 2020). Despite these issues, there is value in using retrospective data to explore epidemiologic associations, especially when evaluated with all existing evidence (i.e. like in a systematic review). For instance, an association found with historical data that cannot be replicated with contemporaneous data might be context specific (i.e. suggesting potential cohort or period effects). That said, our finding was consistent with those of longitudinal studies using more recent data and carried out in other countries (Avontroodt et al., 2020; Kenealy et al., 2007; Shaw et al., 2007). Notwithstanding the importance of the context in which the association was tested, all available evidence (including our study) points to the fact that self-esteem is a complex phenomenon which does not depend on a single aspect and is determined by several other elements in an individual’s life (Kiyak, 2008).

Increases in self-esteem over the follow-up period were positively associated with mothers’ education and negatively associated with baseline self-esteem score. Interestingly, parental social class (the other socioeconomic indicator used in this study) was not associated with adolescents’ change in self-esteem. However, our finding is in line with current evidence suggesting that higher parental education was associated with higher levels of global and specific self-esteem (von Soest et al., 2016). According to the reflective appraisal model, family socioeconomic status (in terms of prestige and importance) may be an important asset which positively influence others’ perception of an individual, which may in turn be internalised to enhance that individual’s own self-worth (Twenge and Campbell, 2002). The negative association between baseline and change in self-esteem score can be explained by the so-called regression to the mean (Barnett et al., 2005), which shows that individuals with extreme values in a continuous measure at baseline are likely to be less extreme in a subsequent assessment (regardless of whether there is an intervention in place or not) (Morton and Torgerson, 2005).

This study has some implications for practice and research. Based on the accumulated evidence on this research area thus far, it seems that self-esteem might not be a reasonable outcome to assess in terms of the impact of orthodontic treatment. Orthodontists must explain the outcomes of orthodontic treatment based on the best available evidence to ensure that the patients do not have unrealistic expectations. As for research, this area would benefit from further longitudinal studies using implicit (indirect) measures of self-esteem, preferably administered over multiple time points during adolescence. It would also be helpful to identify precisely the effect of the type of orthodontic treatment (interceptive, comprehensive, etc.) individuals received.

This study has some limitations. First, some cohort members were excluded from the analysis because of missing data on confounders. Indeed, we found some differences between participants included and excluded from the analysis, especially in terms of participants’ ethnicity and mothers’ education. Therefore, the present findings are not generalisable to the study population. Second, information on orthodontic treatment and perceived need for orthodontic treatment was based on parental reports, which are prone to measurement bias. Questions on orthodontic treatment were posed to parents in present tense, and thus, there is a possibility that the responses did not capture adolescents who had completed orthodontic treatment at the time of asking (especially by age 16 years). This would have underestimated the proportion of adolescents who had undergone orthodontic treatment in this study (8%) and may explain why it was slightly lower to that reported in the 1983 Children’s Dental Health Survey (14%) (Chestnutt et al., 2006). In addition, perceived need for orthodontic treatment was indicated by whether the adolescent had had an orthodontist consultation in the past year, which might not have fully identified all participants with normative need. Third, self-esteem was measured using a reduced version of the LAWSEQ containing 10 of the 16 original items. However, this version was used with adolescents previously (Mak and Fancourt, 2019; Viner and Cole, 2006), and is based on evidence that the scale is unidimensional (Rae et al., 2011). Finally, it is possible that unmeasured confounders, such as psychological or mental health issues, might have influenced the results.

## Conclusion

This six-year longitudinal study provided no evidence for an association between history of orthodontic treatment and changes in self-esteem among British adolescents. Improvements in self-esteem might not be a reasonable outcome to consider when evaluating the impact of orthodontic treatment.

## Supplemental Material

sj-doc-1-joo-10.1177_14653125211006113 – Supplemental material for Is orthodontic treatment associated with changes in self-esteem during adolescence? A longitudinal study Click here for additional data file. Supplemental material, sj-doc-1-joo-10.1177_14653125211006113 for Is orthodontic treatment associated with changes in self-esteem during adolescence? A longitudinal study by Srichitra Vulugundam, Lucas Guimarães Abreu and Eduardo Bernabé in Journal of Orthodontics
