# Supplementary material for: Is orthodontic treatment associated with changes in self-esteem during adolescence? A longitudinal study
Source: J Orthod. 2021 Apr 16;48(4):352–9. doi: 10.1177/14653125211006113 (PMC8652369; doi:10.1177/14653125211006113)
Supplement: sj-doc-1-joo-10.1177_14653125211006113 – Supplemental material for Is orthodontic treatment associated with changes in self-esteem during adolescence? A longitudinal study [file sj-doc-1-joo-10.1177_14653125211006113.doc]

STROBE Statement—checklist of items that should be included in reports of observational studies

|  | Item No | | Recommendation | Page |
| --- | --- | --- | --- | --- |
| **Title and abstract** | 1 | | (*a*) Indicate the study’s design with a commonly used term in the title or the abstract | 1 |
|  |  |  | (*b*) Provide in the abstract an informative and balanced summary of what was done and what was found | 1 |
| Introduction | | | |  |
| Background/rationale | 2 | | Explain the scientific background and rationale for the investigation being reported | 2 |
| Objectives | 3 | | State specific objectives, including any prespecified hypotheses | 3 |
| Methods | | | |  |
| Study design | 4 | | Present key elements of study design early in the paper | 3 |
| Setting | 5 | | Describe the setting, locations, and relevant dates, including periods of recruitment, exposure, follow-up, and data collection | 3 |
| Participants | 6 | | (*a*) *Cohort study*—Give the eligibility criteria, and the sources and methods of selection of participants. Describe methods of follow-up | 3 |
|  |  |  | (*b*) *Cohort study*—For matched studies, give matching criteria and number of exposed and unexposed | N/A |
| Variables | 7 | | Clearly define all outcomes, exposures, predictors, potential confounders, and effect modifiers. Give diagnostic criteria, if applicable | 3/4 |
| Data sources/ measurement | 8* | | For each variable of interest, give sources of data and details of methods of assessment (measurement). Describe comparability of assessment methods if there is more than one group | 3/4 |
| Bias | 9 | | Describe any efforts to address potential sources of bias | 4/5 |
| Study size | 10 | | Explain how the study size was arrived at | 5 |
| Quantitative variables | 11 | | Explain how quantitative variables were handled in the analyses. If applicable, describe which groupings were chosen and why | 4/5 |
| Statistical methods | 12 | | (*a*) Describe all statistical methods, including those used to control for confounding | 4/5 |
|  |  |  | (*b*) Describe any methods used to examine subgroups and interactions | 5 |
|  |  |  | (*c*) Explain how missing data were addressed | 5 |
|  |  |  | (*d*) *Cohort study*—If applicable, explain how loss to follow-up was addressed | 5 |
|  |  |  | (*e*) Describe any sensitivity analyses | N/A |
| Results | | | |  |
| Participants | 13* | (a) Report numbers of individuals at each stage of study—eg numbers potentially eligible, examined for eligibility, confirmed eligible, included in the study, completing follow-up, and analysed | | 5 |
|  |  | (b) Give reasons for non-participation at each stage | | 5 |
|  |  | (c) Consider use of a flow diagram | | No |
| Descriptive data | 14* | (a) Give characteristics of study participants (eg demographic, clinical, social) and information on exposures and potential confounders | | 5 |
|  |  | (b) Indicate number of participants with missing data for each variable of interest | | 5 |
|  |  | (c) *Cohort study*—Summarise follow-up time (eg, average and total amount) | | 3 |
| Outcome data | 15* | *Cohort study*—Report numbers of outcome events or summary measures over time | | *5* |
| Main results | 16 | (*a*) Give unadjusted estimates and, if applicable, confounder-adjusted estimates and their precision (eg, 95% confidence interval). Make clear which confounders were adjusted for and why they were included | | Table 3 |
|  |  | (*b*) Report category boundaries when continuous variables were categorized | | N/A |
|  |  | (*c*) If relevant, consider translating estimates of relative risk into absolute risk for a meaningful time period | | N/A |
| Other analyses | 17 | Report other analyses done—eg analyses of subgroups and interactions, and sensitivity analyses | | 6 |
| Discussion | | | |  |
| Key results | 18 | Summarise key results with reference to study objectives | | 6 |
| Limitations | 19 | Discuss limitations of the study, taking into account sources of potential bias or imprecision. Discuss both direction and magnitude of any potential bias | | 8 |
| Interpretation | 20 | Give a cautious overall interpretation of results considering objectives, limitations, multiplicity of analyses, results from similar studies, and other relevant evidence | | 6 |
| Generalisability | 21 | Discuss the generalisability (external validity) of the study results | | 8 |
| Other information | | | |  |
| Funding | 22 | Give the source of funding and the role of the funders for the present study and, if applicable, for the original study on which the present article is based | | Title page |
